# Water-Closets

**Published:** 1866-03

**Authors:** 


### WATER-CLOSETS.

The location of these, in connection with a family residence, has an important bearing on the health of any family; a greater influence on the destiny of many than would be supposed by other than a medical practitioner, from the operation of a single law of the animal economy, in connection with a fact, to be afterward stated, which no observant person can truthfully deny. It is of the very first importance that the water-closet should be always, and instantly, and easily accessible; in proportion as this is not the case, the calls of nature are postponed. This never can be done with impunity, for nature never does any thing in vain, nor out of time. But it is singular to observe how she never allows herself, as it were, to be trifled with; if her call is not heeded, it is less and less urgent; her appeals to the nerves of sensation are less and less strong, until they cease to be felt; the inclination passes off, and it may be hours before she has recovered strength to call again; but with this unvarying result, the next day the call is made later, and later, and later, until after a while it is omitted for a whole day, and before the person is aware of it, it is found that the bowels are constipated; that several days pass without an evacuation, and with this, certain uncomfortable feelings are observed, entirely new to the person in question; they are simply "symptoms," the indications that disease is setting up in the system; such as headache, cold feet, bad taste in the mouth on getting up in the morning, an irregular appetite, qualmishness, an absence of accustomed vivacity; and in due time there is actual disease, in the shape of sick headache, sour stomach, piles, wasting diarrhea, catarrh, "the least thing in the world gives me a cold," dyspepsia, with all its horrors, or a general decline of the whole system. Every observant physician knows that more than half of all ordinary diseases have their foundations laid in a constipated condition of the bowels—that is, a failure in them to act every day with almost the regularity of the rising of the sun; and he further knows, that the beginning of this irregularity was brought about by deferring the calls of nature until company was gone; until the chapter was finished; until the newspaper was looked over; until some work in hand was completed; or until "the coast was clear." It is in this, as in thousands of other cases, that the greatest of calamities arise sometimes, from almost inappreciable causes; and in all human record there is not a stronger exemplification of it than in the case in hand. There are thousands and tens of thousands of intelligent and observant persons in mature life, and still later on in years, who would cheerfully give a large portion of what they possess if they could have a natural, regular action of the bowels every day, without any artificial aid; and who can and do look back in vain remorses to the times when there was a proper and healthful regularity, and to the occasions and manner of their first breaking into it, simply for the want of a little personal energy, a little self-denial, a small modicum of force of will, which would resolutely, and even impatiently, clear out of its path those trifling, those cob-web obstacles which were in the way of our physical duty, as it were. But it is not always that nature allows persons to escape with a moderate or protracted or slow punishment. There are multitudes of cases recorded where, from motives of false delicacy, as riding in public vehicles, waiting for others, or for daybreak to come, or from sheer laziness, the power to pass water has been taken away, acute inflammation has set in, and death has followed in two or three days. It is well worth while then to be at pains to say all that has been done, if by it a single family should, in the erection of a new house, or in the remodeling of an old one, be led to make a wise and practical use of the facts which have been presented, in having a large privy constructed, with five or six apartments, appropriated to the different classes of the family, so that one may never need have to wait on another for a single instant, and also that approaches may be made with as much privacy as practicable, and by a path protected from the weather, to be used when inclement, and by another to be used in good weather, and still as distant from the house as can be conveniently arranged; for example, to be approached through the wood-house, and also through the garden. The deposits should be made in a water-proof receptacle, placed on the surface of the earth, on runners or wheels, to be removed and emptied once a week on pasture or other land. The debris of one individual will fertilize an acre of ground every year, to an extent greater than any ordinary compost. In addition, for the seven warmer months of the year, lime or fresh ashes of wood should be scattered around the receptacle every fortnight, while a gallon or two of the following solution should be thrown into the receptacle itself every week or two. One pound of copperas, known as "sulphate of iron," costing but a few cents, dissolved in four gallons of water, will most completely destroy all offensive odors, whether in sinks, privies, or cellars. The warmer the weather, the oftener must the application be repeated. Sprinkling the copperas itself about is advantageous, and, if in cellars, is one of the best means of keeping rats away.

It is advisable to have a water-closet in some convenient part of every dwelling, to be used only on emergencies, which may occur during the night, for example.

One of the happiest thoughts in this connection, and one which could scarcely occur to any other than one of the members of the Society of Friends, so remarkable for their thoughtfulness and happy talent of having about them all the conveniences and appliances which so much add to the comforts and enjoyments of domestic life, was in having a water-closet connected with his barn, for the convenience of his gentlemen friends who visit him in the summer at his delightful mansion on the banks of the Hudson; this is one of the earliest pieces of information given to those coming for the first time; to this they can repair at any hour, with a feeling of perfect privacy.

